# Association between age at menarche and infertility in Tabari cohort population: a population-based case-control study

**DOI:** 10.1186/s13104-025-07357-2

**Published:** 2025-07-09

**Authors:** Marzieh Zamaniyan, Mina Esmaeili, Sepideh Peivandi, Shamim Khorshidian, Mobina Gheibi, Monirolsadate Hosseini Tabaghdehi, Mahmood Moosazadeh, Erfan Ghadirzadeh

**Affiliations:** 1 Department of Obstetrics and Gynecology, Sexual and Reproductive Health Research center, School of Medicine, Mazandaran University of Medical Sciences, Sari, Iran; 2 Student Research Committee, School of Medicine, Mazandaran University of Medical Sciences, Sari, Iran; 3 Gastrointestinal Cancer Research Center, Non-Communicable Diseases Institute, Mazandaran University of Medical Sciences, Sari, Iran; 4 Department of Midwifery, Islamic Azad University, Sari, Iran; 5 Department of Laboratory Sciences, Razi Hospital, Mazandaran University of Medical Sciences, Qaemshahr, Iran

**Keywords:** Age at menarche, Infertility, Fertility, Menstruation, Reproductive, Iran

## Abstract

**Objective:**

There are conflicting results regarding the association between age at menarche (AAM) and infertility in different nations. Thus, this study aimed to determine this relationship in a cohort of northern Iranian population (Tabari Cohort Study).

**Results:**

The mean menarche age for the case and control groups was 13.48 ± 1.63 and 13.67 ± 1.72, respectively (P: 0.060). The rate of infertility in women of early, normative, and late menarche were 56.3%, 50.4% and 30.4% respectively. The results of the adjusted logistic regression analysis showed that women with early AAM (OR: 3.36, 95% CI: 1.48–7.63, P: 0.004) and normative AAM (OR: 2.90, 95% CI: 1.42–5.90, P: 0.003) had higher odds for infertility compared to females with late menarche with a trend toward higher risk in earlier AAM.

## Background

Infertility is defined as the failure to conceive within one year of regular unprotected intercourse and leads to marital dissatisfaction, possible domestic violence, and reduced birth rates, with an estimated global prevalence at 12.87% and 10.5% in Iran [1–4]. Female fertility is related to a series of important biological reproductive stages, including menarche, menstruation, pregnancy, breastfeeding, and menopause [5]. Menarche marks the beginning of the reproductive cycle. In recent years, many populations have seen a trend toward earlier or later onset of the first menstrual cycle in girls. This change has sparked concerns about its potential long-term effects, especially on fertility [6].

Studies on the association between age at menarche (AAM) and infertility have shown conflicting results. Some studies have shown that infertility rates are higher in people with an earlier AAM [7–9], whereas others have shown that later AAM is associated with infertility [10, 11].

Within the Iranian context specifically, the existing literature on the AAM-infertility relationship is notably scarce and contradictory. A case-control study by Delpishe et al. [12] involving women in Ilam province reported significantly increased odds of both primary and secondary infertility for both early menarche (OR: 10.42, 95% CI: 3.06–35.49) and late menarche (OR: 1.98, 95% CI: 1.27–3.10) compared to normative menarche. In contrast, a more recent nationwide cross-sectional study by Jenabi et al. [13] found no significant association between late menarche and primary infertility (OR: 1.34, 95% CI: 0.72–2.53) and suggested a potential protective effect of early menarche (OR: 0.57, 95% CI: 0.35–0.93), though definitions and reference groups differed. This inconsistency underscores a significant gap in the Iranian literature. Consequently, robust population-based evidence from a well-defined cohort is critically needed to clarify the association within the unique sociodemographic and environmental context of Northern Iran.

Nevertheless, while the results of several studies have shown that AAM is associated with infertility [7–11, 14], other researchers believe that the timing of menarche alone is not a predictor of infertility, but that other factors, including body mass index (BMI), lifestyle, stress, smoking, socioeconomic status, and exposure to environmental toxins, such as endocrine-disrupting chemicals, play a more pivotal role [15].

Given these limitations and inconsistencies regarding the effect of AAM on fertility, we aimed to investigate the relationship between AAM and infertility in a cohort of northern Iranian population.

## Materials and methods

### Population and study design

This study was a case-control study conducted in the Tabari cohort study (TCS) population. The TCS itself is part of a national mega cohort, the PERSIAN cohort. The current study was conducted based on data from the TCS enrollment phase conducted between 2015 and 2017, with a total of 10,255 enrolled participants aged 35–70 years (6103 females). The details and characteristics of the standard questionnaires used for TCS data collection are described in the cohort’s methodological papers [16, 17]. The case group included 488 infertile women selected through census. The control group was randomly selected from fertile women in the same sample size.

### Inclusion/exclusion criteria

#### Case

All infertile females (failure to conceive within one year of regular unprotected intercourse) were included using census sampling. Patients with surgically induced infertility (e.g. hysterectomy) or other causes of secondary infertility, congenital reproductive system anomaly, or male partner infertility were excluded.

#### Control

Fertile females (either pregnant at the moment or had pregnancy before) were randomly selected in a 1:1 ratio from the pool of eligible fertile women in the same cohort without matching, with confounders addressed analytically rather than through study design. Females that had conceived using Assisted Reproductive Technologies (e.g. in vitro fertilization, intrauterine insemination) were excluded.

### Measurements

In the present study, demographic information, including age, residential area (urban or rural), education level, occupation, socioeconomic status (SES), chronic diseases such as thyroid disorders (TD) and diabetes mellitus (DM), level of physical activity (PA) measured in metabolic equivalent of task (MET), and anthropometric measurements, including BMI, waist-to-hip ration (WHR), and waist circumference (WC), was obtained from the TABARI cohort data repository. Reproductive data including AAM, oral contraceptive (OCP) use, history of abortion, and age at first marriage, were also extracted from the TCS database. For the purpose of this study, participants were classified into three groups based on their AAM: early menarche (< 12 years), normative menarche (12–16 years), and late menarche (> 16 years).

SES was derived using Principal Component Analysis (PCA) on asset-based variables: domestic/international travel frequency, reading habits, computer/internet access, vehicle ownership (car/motorcycle), housing status (owner/tenant), and household appliance ownership (dishwasher, washing machine, freezer, vacuum cleaner). The first principal component (explaining 58% of variance) was divided into quintiles to create an ordinal SES index: Level I (lowest 20%), Level II (next 20%), Level III (middle 20%), Level IV (next 20%), and Level V (highest 20%).

PA was quantified in METs using the validated PERSIAN cohort questionnaires. Activities were coded via the Compendium of Physical Activities [18]. Participants were dichotomized as “Higher PA” (≥ median MET-min/week) or “Lower PA” (< median MET-min/week) based on cohort-specific distribution.

### Statistical analysis

Data analysis was performed using SPSS software version 26. Mean, standard deviation (SD), and frequencies were used to describe the variables. Chi-square and T-test were used to compare qualitative and quantitative variables between the two groups. Univariate and multiple logistic regression analyses were used to adjust the effect of confounding variables (age, residential area, educational level, PA level, occupational status, age at first marriage, OCP use, and history of abortion) in this study to assess the association between AAM and infertility (variables with a P-value of less than 0.250 in the Univariate model were included in the multivariate regression model). The odds ratio (OR) was used to examine the odds of infertility with the 95% confidence interval (95%CI).

## Results

In the present study, 488 infertile women (case) and 488 non-infertile women (control) were studied. The mean menarche age for the case and control groups was 13.48 ± 1.63 and 13.67 ± 1.72, respectively (P: 0.06), but when categorized, infertile females tended to have earlier AAM. As demonstrated in Table 1, out of 488 infertile females, 54 (56.3%) had early menarche, 420 (50.4%) had normative menarche, and 14 (30.4%) had late menarche, whereas among fertile females, 42 (43.8%) had early, 414 (49.6%) had normative, and 32 (69.6%) had late menarche (P: 0.014). Infertile females were more likely to live in city than controls (52.5% vs. 47.5%, P: 0.009), had married at older ages (*P* < 0.001), had taken less OCPs (39.9% vs. 60.1%, *P* < 0.001), but had higher rated of abortion history (59.9% vs. 40.1%, *P* < 0.001). There was no statistically significant difference between the two groups in terms of educational level, SES, PA level, occupation, TD, DM, smoking, BMI, WHR, or WC (all *P* > 0.05) (Table 1).

**Table 1:** Baseline characteristics including demographic variables, underlying conditions and anthropometric measures of the study groups

Variables		Case: *n*, (%)	Control: *n*, (%)	*P*-value
Age	35–39	111 (54.7%)	92 (45.3%)	0.001
	40–49	202 (55.5%)	162 (44.5%)	
	50–59	120 (45.6%)	143 (54.4%)	
	60–70	55 (37.7%)	91 (62.3%)	
Residence	Urban	374 (52.5)	338 (47.5)	0.009
	Mountainous	114 (43.2)	150 (56.8)	
Education	University	91 (52)	84 (48)	0.108
	9–12 years in school	142 (49.7)	144 (50.3)	
	6–8 years in school	54 (57.4)	40 (42.6)	
	1–5 years in school	128 (52)	118 (48)	
	No schooling	73 (41.7)	102 (58.3)	
SES	1 (Lowest)	87 (43.5%)	113 (56.5%)	0.270
	2	107 (54.0%)	91 (46.0%)	
	3	105 (52.5%)	95 (47.5%)	
	4	102 (50.0%)	102 (50.0%)	
	5 (Highest)	87 (50.0%)	87 (50.0%)	
PA	< Median	285 (52.4%)	259 (47.6%)	0.094
	≥ Median	203 (47.0%)	229 (53.0%)	
Occupation	Employed	76 (44.7)	94 (55.3)	0.129
	Unemployed	412 (51.1)	394 (48.9)	
Age at first marriage	< 16	46 (41.1)	66 (58.9)	< 0.001
	16–25	338 (47.9)	368 (52.1)	
	26–35	71 (58.7)	50 (41.3)	
	> 35	33 (89.2)	4 (10.8)	
TD	Yes	77 (52.7)	69 (47.3)	0.473
	No	411 (49.5)	419 (50.5)	
DM	Yes	91 (50.8)	88 (49.2)	0.804
	No	397 (49.8)	400 (50.2)	
Smoking	Yes	5 (62.5)	3 (37.5)	0.725
	No	483 (49.9)	485 (50.1)	
BMI	< 25	84 (48)	91 (52)	0.509
	25-29.9	175 (48.5)	186 (51.5)	
	30 ≤	229 (52)	211 (48)	
WHR	> 0.85	355 (49.4%)	363 (50.6%)	0.561
	≤ 0.85	133 (51.6%)	123 (48.4%)	
WC	< 88	147 (50.0%)	147 (50.0%)	1.000
	≥ 88	341 (50.0%)	341 (50.0%)	
OCP	Yes	148 (39.9)	223 (60.1)	< 0.001
	No	340 (56.2)	265 (43.8)	
Had Abortion	Yes	269 (59.9)	180 (40.1)	< 0.001
	No	219 (41.6)	308 (58.4)	
AAM	< 12	54 (56.3%)	42 (43.8%)	0.014
	12–16	420 (50.4%)	414 (49.6%)	
	> 16	14 (30.4%)	32 (69.6%)	

SES: Socioeconomic Status, PA: Physical Activity, TD: Thyroid Disorder, DM: Diabetes Mellitus, BMI: Body Mass Index, WHR: Waist-to-Hip Ration, WC: Waist Circumference, OCP: Oral Contraceptive, AAM: Age at Menarche

Univariate logistic regression showed that both early AAM (OR: 2.93, 95%CI: 1.39–6.19, P: 0.005) and normative AAM (OR: 2.31, 95%CI: 1.22–4.40, P: 0.010) had higher odds of infertility compared to women with late AAM (P for trend: 0.014) showing higher risk of infertility with earlier menarche age (OR: 2.93 vs. 2.31) (Table 2). Similarly, the multiple regression model also demonstrated that both early menarche (OR: 3.36, 95%CI: 1.48–7.63, P: 0.004) and normative menarche (OR: 2.90, 95%CI: 1.42–5.90, P: 0.003) had higher odds of infertility compared late menarche, with increased odds after adjustment for confounders (compared to the Univariate analysis), and a trend toward higher risk in earlier menarche age (OR: 3.36 vs. 2.90) (Table 2).

**Table 2:** Investigating factors associated with infertility based on multiple logistic regression

Variables		Univariate logistic regression			Multiple logistic regression*		
		OR	95%CI	P-value	OR	95%CI	P-value
AAM	< 12	2.93	1.39–6.19	0.005	3.36	1.48–7.63	0.004
	12–16	2.31	1.22–4.40	0.010	2.90	1.42–5.90	0.003
	> 16	Ref	Ref	Ref	Ref	Ref	Ref

*Adjusted for age, residential area, educational level, physical activity level, occupational status, age at first marriage, OCP use, and history of abortion

## Discussion

In the present study, we investigated the relationship between menarche age and infertility in the TCS. 488 infertile women and 488 non-infertile individuals were included in the study. The odds of infertility among females with early and normative menarche was significantly higher than females with late menarche, with a trend toward higher risk in earlier AAM.

Early menarche has been associated with various adverse health outcomes such as cancers, cardiovascular diseases, miscarriages and premature death [19]. A Korean study linked early menarche to a higher risk of premature ovarian insufficiency and idiopathic early menopause. These findings support the current study’s observation that late menarche may be protective against infertility [20].

To elucidate the trend observed in our study, where earlier menarche corresponds to higher infertility risk, we explored potential biological mechanisms that may underlie this association: Early menarche is a recognized risk factor for PCOS, a leading cause of infertility due to ovulatory dysfunction. A Mendelian randomization study by Ma et al. [9] demonstrated that earlier AAM was causally associated with an increased risk of PCOS. The earlier onset of menstrual cycles may amplify hormonal disruptions, contributing to PCOS development and thus elevating infertility risk. Early menarche may lead to diminished ovarian function or a faster depletion of ovarian reserve. Weghofer et al. [21] found that women with early menarche (< 13 years) exhibited reduced ovarian function compared to those with later menarche (≥ 13 years). This could result from an accelerated loss of oocytes, shortening the fertile window and increasing infertility risk, particularly in women with earlier menarche. Early menarche has been linked to premature menopause (before age 40) and early menopause (ages 40–44), which shorten the reproductive lifespan. Mishra et al. [8] reported that women with early menarche (≤ 11 years) had a higher risk of premature and early menopause. This mechanism may explain the gradient of infertility risk in our study, as earlier menarche could lead to an earlier cessation of reproductive capacity. Also, Early menarche increases the risk of endometriosis, a condition associated with infertility due to pelvic adhesions and ovulatory dysfunction. A systematic review and meta-analysis by Nnoaham et al. [22] confirmed that early menarche was a significant risk factor for endometriosis. The longer duration of menstrual cycling in women with early menarche may exacerbate this risk, contributing to the observed trend.

Studies have reported conflicting results regarding AAM and fertility. For instance, a study by Ban et al. [6] found that each one-year increase in AAM corresponded with a 16.7% increase in the risk of polycystic ovary syndrome (PCOS) among women with primary infertility, indicating that late menarche age may be associated with endocrine-related infertility causes. Conversely, Mishra et al. [8] reported that early menarche (≤ 11) was a risk factor for both premature menopause (final menstrual period before age 40) and early menopause (final menstrual period between ages 40–44), suggesting that early menarche could lead to a shortened reproductive lifespan.

Studies have suggested various reasons for the association of infertility with early menarche. One of the proposed reasons is that early menstruation is associated with poor reproductive function, including irregular periods, PCOS, and an increased risk of endometriosis [8]. In this regard, the results of the study by Weghofer et al. [21] showed that ovarian function was reduced in women with early menarche (< 13) compared to women with late menarche (≥ 13).

Another reason is the increased rate of miscarriage in women with early menarche. One cause of miscarriage is chromosomal abnormalities [23]. Lu Zhao [24] reported that the frequency of women with a history of miscarriage who had abnormal fetal chromosomes was higher among women with a menarche age of less than 12 years than among women with a menarche age of more than 12 years.

In contrast, a cross-sectional study in China on 6906 couples showed that increasing AAM was associated with an increased risk of infertility. The lowest frequency of infertility was observed in AAM of less than 12 years (5.75%) and the highest frequency was observed in AAM of over 18 years (17.29%) [25]. The results of this study are in contrast to the results of the present study. The large sample size, racial differences, and the retrospective nature of the study may also explain the discrepancy in results.

In the present study, infertile participants were married at an older age. In many countries, such as Iran, fertility is largely restricted to marriage due to cultural reasons, and marriage itself marks the beginning of exposure to pregnancy. For example, in India, where most births occur after marriage, and age at marriage influences the number of children a woman ultimately has. Thus, increasing age at marriage may lead to a decline in fertility by shortening the fertile period [26].

Additionally, fertile participants had higher history of OCP use in the present study. The progestin in OCPs thickens the cervical mucus, which is resistant to bacterial penetration. This contraceptive mechanism reduces the risk of upper genital tract infections. Epidemiological studies have shown a significant reduction in the risk of PID associated with OCP use [27]. OCPs also treat endometriosis, a cause of infertility [28]. On the other hand, women who have had a history of childbearing use OCPs as a method of preventing further pregnancies, which could be a possible explanation for the higher frequency of use of these drugs compared to women with infertility.

In the present study, no differences were observed in anthropometric measurements, but it should be noted that these measurements were recorded during the enrollment phase of the TCS, and individuals may have been obese/overweight/normal weight at other times in their lives.

### Limitations

One limitation of the study is the potential inaccuracy in reporting the AAM due to recall bias. Since menarche often occurs years before data collection, participants may struggle to remember the exact age at which it happened. This reliance on self-reported retrospective data can lead to misclassification, where some individuals may over- or underestimate their AAM. However, given menarche’s significance as a major developmental milestone in Iranian culture, comparable to marriage or first childbirth in memorability, participants are likely to recall this event with reasonable accuracy. Future studies could mitigate this by: [1] collecting AAM prospectively in longitudinal cohorts starting pre-menarche; [2] using anchored recall methods (e.g., linking to historical/educational milestones); [3] validating self-reports against early medical records where available; and [4] implementing computer-assisted interviews with visual timelines. Also, validation in cohorts with varying genetic backgrounds (e.g., African, European) and environmental exposures is warranted. Future studies should explicitly test whether our effect size (OR: 3.36 for early menarche) varies by geographic setting to assess to what extent these findings are generalizable to other populations. Finally, the limited number of late-menarche women (*n* = 46) reduced precision for this subgroup.

## Conclusion

The odds of infertility among females with early and normative menarche was significantly higher than females with late menarche, with a trend toward higher risk in earlier AAM.

## Data Availability

The data are available upon reasonable request from the corresponding author.
